# Short-Term Clinical Implications of the Accessory Left Hepatic Artery in Patients Undergoing Radical Gastrectomy for Gastric Cancer

**DOI:** 10.1371/journal.pone.0064300

**Published:** 2013-05-23

**Authors:** Chang-Ming Huang, Qi-Yue Chen, Jian-Xian Lin, Chao-Hui Zheng, Ping Li, Jian-Wei Xie, Jia-Bin Wang, Jun Lu

**Affiliations:** Department of Gastric Surgery, Fujian Medical University Union Hospital, Fuzhou, Fujian Province, China; National Cancer Center, Japan

## Abstract

**Background:**

To evaluate the prevalence of the accessory left hepatic artery (ALHA; defined as a vessel arising from the left gastric artery, which, together with a typical left hepatic artery, supplies blood to the left lobe of the liver) and its short-term clinical implications in patients undergoing radical gastrectomy for gastric cancer.

**Methods:**

Clinical data of 1173 patients with gastric cancer who underwent laparoscopy-assisted radical gastrectomy were retrospectively analyzed. Groups of patients with and without ALHA were compared to identify differences in intraoperative and postoperative variables and changes in liver function.

**Results:**

Of the 1173 patients, 135 (11.5%) had an ALHA and 1038 (88.5%) did not. There were no significant between-group differences in clinicopathological and intraoperative characteristics, postoperative recovery, and morbidity and mortality rates (P>0.05 each). None of the patients had postoperative symptoms associated with impaired liver function. Glutamic oxaloacetic transaminase (GOT), glutamic pyruvic transaminase (GPT) and total bilirubin (TBIL) concentrations were similar preoperatively. TBIL concentrations on postoperative days 1, 3, and 7 were similar (P>0.05), while GOT and GPT activities were higher in the ALHA than in the non-ALHA group on days 1 and 7 (P<0.05), with all three markers similar in the two groups on day 14. In patients without chronic liver disease (CLD), GOT, GPT and TBIL concentrations were similar in patients with and without ALHA; whereas, in patients with CLD, GOT and GPT concentrations on days 1 and 3 and GOT on day 7 were higher in patients with than without ALHA.

**Conclusion:**

ALHA is a common anomaly that was found in 11.5% of patients. It can be safely severed during radical gastrectomy in patients without CLD, but should be left intact in patients with CLD to prevent liver dysfunction. If severed in the latter, the patient should be monitored and liver-protecting therapy may be necessary.

## Background

Japanese guidelines for the treatment of gastric cancer indicate the need for complete removal of the gastrohepatic ligament during radical gastrectomy for gastric cancer [1]. Vascular variations, however, are frequently encountered [2], including an accessory left hepatic artery (ALHA) and accessory left gastric artery, with the ALHA showing the highest incidence [3], [4]. Although variations in the hepatic artery have been assessed by medical imaging and anatomic methods, as well as in patients undergoing liver transplantation or transcatheter arterial chemoembolization (TACE)[5]–[9], few large studies have evaluated the prevalence of ALHA and its impact on patients undergoing radical gastrectomy for gastric cancer. We therefore retrospectively evaluated clinical data of 1173 gastric cancer patients who underwent laparoscopy-assisted radical gastrectomy to determine the prevalence of ALHA and its short-term clinical implications in these patients.

## Materials and Methods

### Patients

Between May 2007 and February 2012, 1173 patients underwent laparoscopy-assisted radical gastrectomy for gastric cancer at Fujian Medical University Union Hospital, with all operations performed by the same group of gastric surgeons. All data were collected from a “clinical data mining system for gastric cancer surgery”[10] and a video data system.

Before surgery, all patients were examined by multidetector computed tomography (MDCT) to evaluate the tumors and their relationship with peripheral vascular structures.The presence or absence of an ALHA was assessed intraoperatively, and patients were divided into groups, with and without ALHA. Intraoperative and postoperative variables and changes in liver function were compared in the two groups. Patients with chronic liver disease (CLD) were defined as those with[11]: (1) at least two abnormal results on tests of glutamic oxaloacetic transaminase (GOT), glutamic pyruvic transaminase (GPT) or total bilirubin (TBIL), at least 6 months apart; (2) imaging results showing radiological signs of cirrhosis and portal hypertension, or a hepatic mass, and evidence of CLD; (3) a liver biopsy consistent with CLD; or (4) a previous diagnostic clinical event (e.g. variceal bleed, spontaneous bacterial peritonitis, or ascites). All patients were stratified by the presence or absence of CLD. Patients were staged according to the tumor, node, metastasis (TNM) classifications of the 7th edition of the Union for International Cancer Control (UICC) [12]. Liver function was assessed by measuring changes over time in GOT, GPT, and TBIL concentrations. Though meticulous preoperative preparation all our patients before surgery are A level according to Child-Pugh classification.

All surgical procedures were performed after obtaining informed consent from each patient. Patients were included if they had histologically confirmed adenocarcinoma of the stomach; no evidence of distant metastasis (e.g., in the liver or lungs) or para-aortic lymph node involvement during preoperative examination; and had undergone R0 radical gastrectomy, as assessed postoperatively. Patients were excluded if they had confirmed stage T4b tumors or intraoperative evidence of peritoneal disseminated or distant metastasis; or incomplete clinicopathological data.

### Ethics Statement

Ethics committee of Fujian union hospital approved this retrospective study (Approval number: 20070428). Written consent was given by the patients for their information to be stored in the hospital database and used for research.

### Surgical procedure

**Figure 1:**
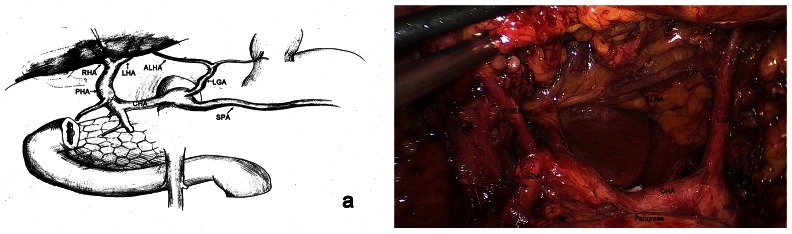
Schematic drawing of the accessory left hepatic artery (ALHA). (**a**). Intraoperative photograph showing an ALHA arising from the left hepatic artery and entering the left liver. (b). LGA, left gastric artery; SPA, splenic artery; CHA, common hepatic artery; PHA, proper hepatic artery; GDA, gastroduodenal artery; RGEA, right gastroepiploic artery; LHA, left hepatic artery; RHA, right hepatic artery.

The type of surgical resection (i.e. distal subtotal gastrectomy, proximal subtotal gastrectomy or total gastrectomy) was selected based on tumor location. Lymphadenectomy was performed in all patients according to the Japanese Gastric Cancer Association [1]. During surgery, all of the tissues around the common hepatic, proper hepatic, celiac axis and splenic arteries were meticulously cleared to remove perigastric lymph nodes, to expose the root of the left gastric artery. If any vessels were encountered during complete resection of the hepatogastric ligament along the inferior border of the liver, fat and lymphoid tissue above and below the vessel was dissected to bare the vessel. If identified, the vessel was dissected along its entire course (up to the hepatic parenchyma and down to its origin from the left gastric artery). Subsequently, the stomach was lifted upwards (towards the head). Fatty connective tissue and lymph nodes along the left gastric artery were thoroughly removed from its root to its access in the stomach to bare the artery. During these procedures, we attempted, whenever possible, to preserve the main vessels and structures and their anatomical relationships to each other. The origin of the ALHA and its course were identified intraoperatively and on preoperative CTA images (Fig. 1), which were carefully recorded onto our video data system by the surgeons. After firmly clipping the ALHA, it was severed at the inferior border of the liver. Finally, the left gastric artery was divided at its origin with double clips to completely remove the lymph nodes (group 7).

### Statistical analysis

All statistical analyses were performed using the statistical program SPSS 18.0. Data were reported as mean ±SD and compared using the chi-square test or unpaired Student's*t*-test, as appropriate. *P*<0.05 was considered statistically significant.

## Results

### 1. Anatomy and prevalence of the ALHA

**Figure 2:**
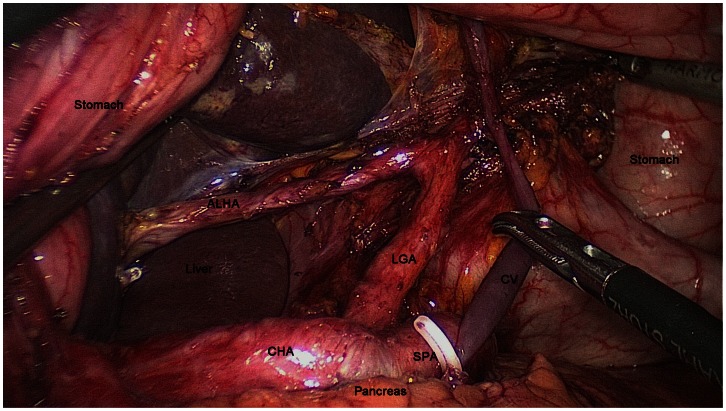
Intraoperative exposure of the origin and terminus of the accessory left hepatic artery (ALHA) though meticulous dissection. LGA, left gastric artery; SPA, splenic artery; CHA, common hepatic artery; CV, coronary vein.

**Figure 3:**
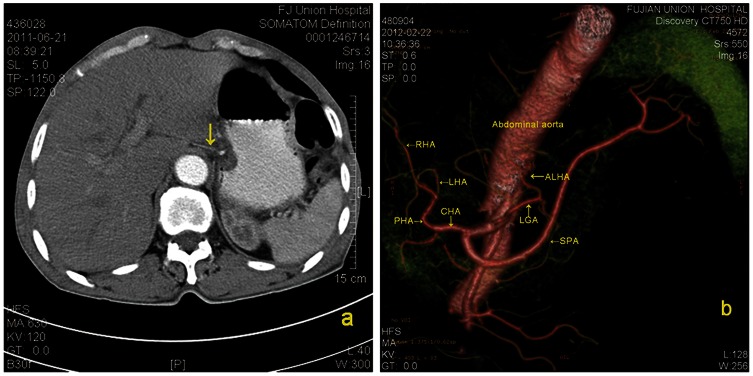
Preoperative enhanced transverse CT image showing a fine accessory left hepatic artery (ALHA) in the gastrohepatic ligament. (**a**). Three-dimensional CT reconstruction,showing an ALHA originating from the left gastric artery. (b). LGA, left gastric artery; SPA, splenic artery; CHA, common hepatic artery; PHA, proper hepatic artery; LHA, left hepatic artery; RHA, right hepatic artery.

**Figure 4:**
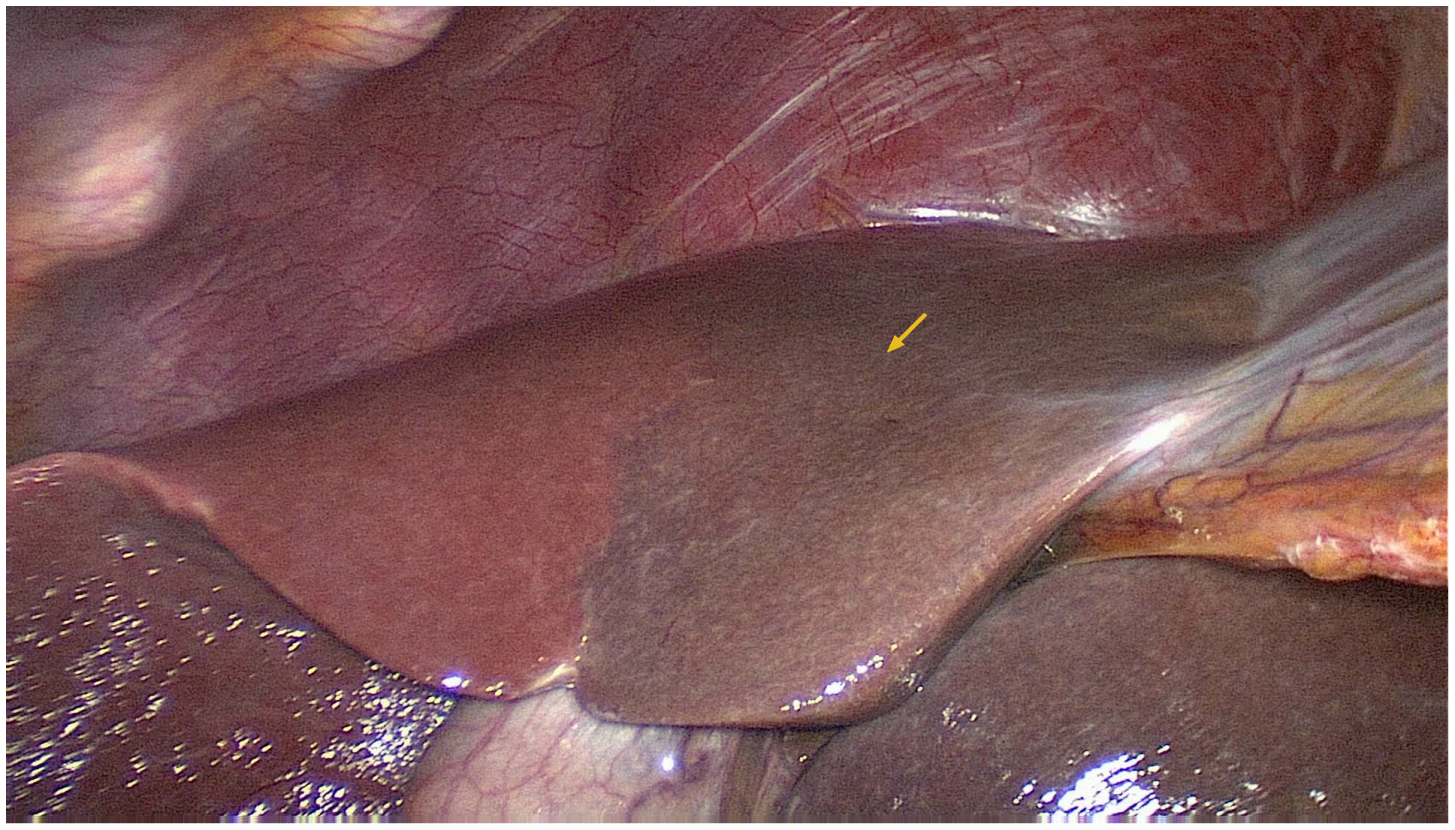
Image showing impairment of the blood supply to the left hepatic parenchyma after a relatively large accessory left hepatic artery (ALHA) was severed (arrows).

The ALHA has been defined as a vessel arising from the left gastric artery, which, in combination with a typical left hepatic artery, supplies blood to the left lobe of the liver [4]. Surgery allows a comprehensive evaluation of the ALHA owing to the meticulous dissection required to trace its origin and terminus, as well as good visualization after the stomach is lifted upwards (Fig. 2). Combining intraoperational finding with CTA images (Fig. 3) enabled a more complete description of the anatomy of the ALHA. The ALHA originates from the left gastric artery, branching off from this artery at its highest or turning point before it runs down toward the lesser curvature of the stomach. The course of the left gastric artery continues toward the upper-left, finally dividing into several branches near the cardia to supply the cardia and the fundus of the stomach. The part of the ALHA outside the liver is short. Within the gastrohepatic ligament, the ALHA extends towards the right or upper-right in a straight or slightly tortuous course to enter the hepatic parenchyma through the left sagittal groove, anterior to the caudate lobe. It extends to the left, dividing into several branches, with the diameters of segmental or subsegmental arteries, to supply the left lateral lobe of the liver. We found that severing a relatively large ALHA intraoperatively resulted in hepatic ischemia, but that the ischemic parenchyma was limited and had clear boundaries separating ischemic from normal parenchyma (Fig. 4).

Of our 1173 patients who underwent radical gastrectomy for gastric cancer 135 (11.5%) had an ALHA, including 107 of the 979 (10.9%) patients without CLD and 28 of the 194 (14.4%) with CLD, a difference that was not statistically significant(P = 0.162).

### 2. Patient clinicopathologic characteristics

The clinicopathologic characteristics of the patients are presented in Table 1. The 1173 patients included 891 men and 282 women, of mean age 60.9 years (range 12 to 101 years). Age, gender, tumor size, body mass index (BMI), location of neoplasm, tumor depth, total number of harvested lymph nodes, lymph node status (N stage), TNM stage, histologic type, resection extent, and gastrointestinal reconstruction type did not differ between the groups of patients with and without an ALHA (*P*>0.05 each).

**Table 1:** Comparison of clinicopathological characteristics in groups of patients with (ALHA group) and without (non-ALHA group) an accessory left hepatic artery (ALHA).

Characteristics	ALHA group(n = 135)	non-ALHA group(n = 1038)	*P* value
Sex			0.831
Female	31	251	
Male	104	787	
Age(years)	60.6±12.4	61.0±11.5	0.721
Tumor size(cm)	5.1±2.8	4.9±2.7	0.507
BMI(kg/m^2^)	21.8±2.9	22.2±3.4	0.175
Tumor location			0.641
Upper	34	264	
Middle	33	290	
Lower	68	484	
Tumor depth			0.559
T1	28	247	
T2	34	228	
T3	31	254	
T4a	42	309	
Total retrieved lymph nodes	30.7±10.8	32.4±10.7	0.104
N stage			0.405
N0	46	384	
N1	25	153	
N2	18	180	
N3	46	321	
TNM stage			0.608
IA	23	209	
IB	16	83	
IIA	12	99	
IIB	13	135	
IIIA	17	119	
IIIB	25	200	
IIIC	29	193	
Histology			0.285
Differentiated	21	210	
Undifferentiated	114	825	
Resection extent			0.942
TG	70	531	
PG	3	28	
DG	62	479	
Reconstruction			0.924
BillrothI	56	420	
BillrothII	6	59	
Roux-en-Y	70	531	
EGP	3	28	

BMI, Body mass index; TG, total gastrectomy; PG, proximal subtotal gastrectomy; DG, distal subtotal gastrectomy; EGP, esophagogastrostomy. *P*-values are for comparison of the ALHA and non-AHLA groups.

### 3. Intraoperative and postoperative characteristics

Operation time, estimated blood loss, volumes transfused, first ambulation time, bowel function recovery time and duration of hospital stay were similar in patients with and without an ALHA (*P*>0.05each; Table 2).

**Table 2:** Comparison of intraoperative and postoperative characteristics in groups of patients with (ALHA group) and without (non-ALHA group) an accessory left hepatic artery (ALHA).

Variables	AHLA group(n = 135)	non-AHLA group(n = 1038)	*P* value
Operation time(minutes) time(minutes)time(minutes)	204.0±379.8	185.3±45.6	0.567
Blood loss(ml)	83.7±87.4	81.64±108.7	0.829
Transfused patients	1	36	0.113
Time to first ambulation(d)	2.5±1.0	2.5±1.1	0.387
Time to first flatus(d)	3.9±1.7	3.7±1.3	0.251
Time to fluid die(d)	4.6±2.0	4.6±1.8	0.959
Time to soft die(d)	8.4±2.7	8.7±3.1	0.148
Hospital stay(d)	13.7±7.1	13.6±8.4	0.853

d represents postoperative days. *P*-values are for comparison of the ALHA and non-AHLA groups.

### 4. Morbidity and mortality

The overall postoperative morbidity and mortality rates among all patients were 11.0% and 0.6%, respectively. Postoperative complication rates (11.1% vs. 10.9%, *P*>0.05) and mortality rates (0.7% vs. 0.6%, *P*>0.05) did not differ between the ALHA and non-AHLA groups (*P*>0.05; Table 3). None of the patients in either group died of liver failure.

**Table 3:** Comparison of morbidity and mortality in groups of patients with (ALHA group) and without (non-ALHA group) an accessory left hepatic artery (ALHA).

Variables	AHLA group(n = 135)	non-ALHA group(n = 1038)	*P* value
Surgical	10	69	0.740
Duodenal stump fistula	1	4	
Anastomotic leakage	2	12	
Pancreatic fistula	1	8	
Lymphatic fistula	1	10	
Abdominal infection	2	10	
Gastric stasis	2	13	
Anastomotic bleeding	1	9	
Anastomotic stenosis	0	3	
Medical	5	45	0.733
Pneumonia	4	35	
Angiocardiopathy	0	8	
DIC	1	2	
Mortality	1	6	0.859

DIC, disseminated intravascular coagulation. *P*-values are for comparison of the ALHA and non-ALHA groups.

### 5. Changes in Liver Function

**Figure 5:**
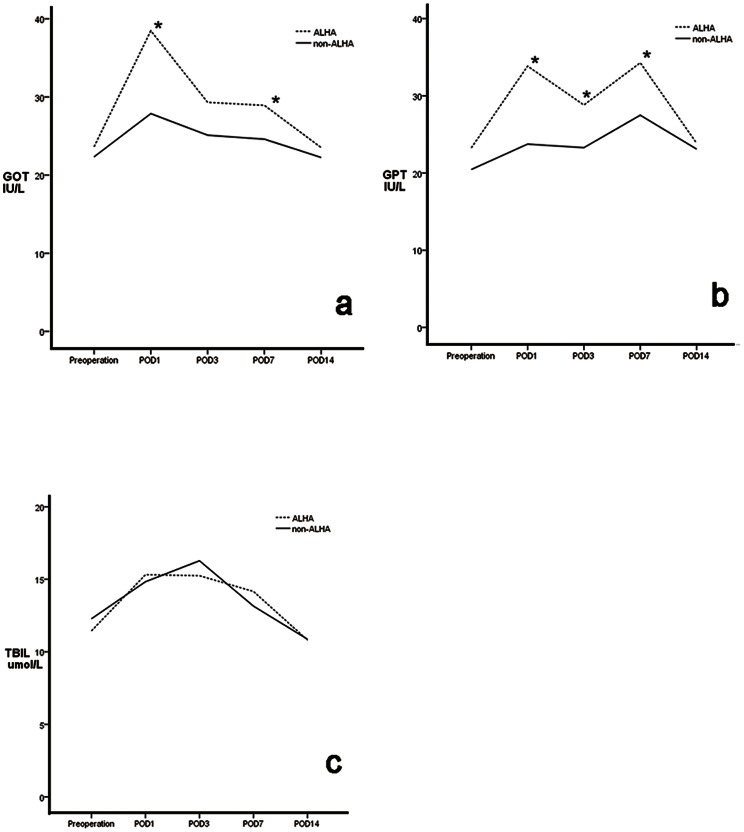
Mean GOT (a), GPT (b), and TBIL (c) concentrations in groups of patients with (ALHA group) and without (non-ALHA group) an accessory left hepatic artery (ALHA). Each value represents the mean.* *P*<0.05.

**Figure 6:**
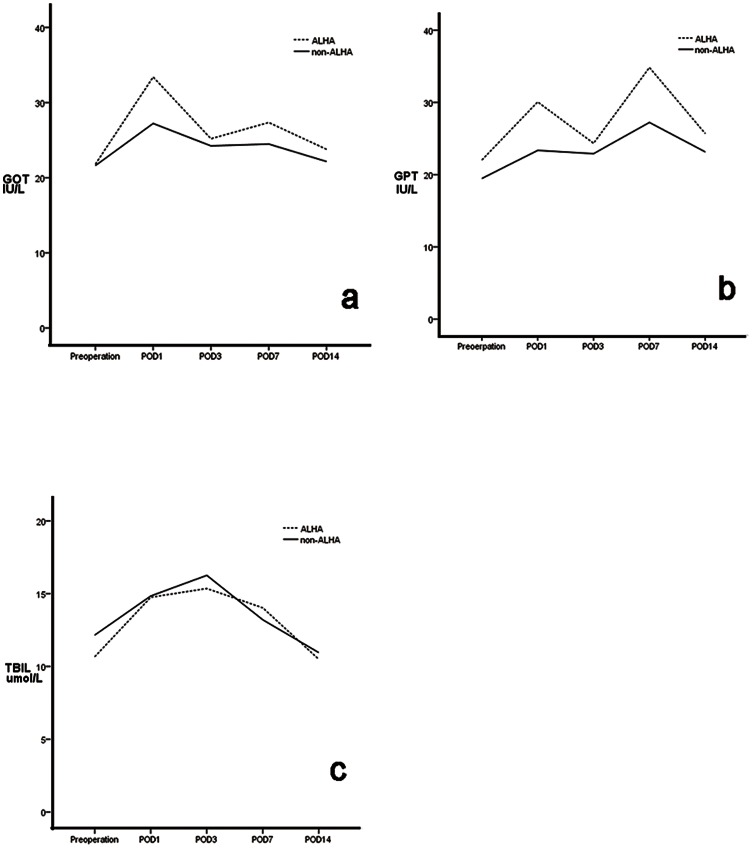
Mean GOT (a), GPT (b), and TBIL (c) concentrations in groups of patients without chronic liver diseases (CLD), with (ALHA group) and without (non-ALHA group) an accessory left hepatic artery (ALHA). Each value represents the mean.

**Figure 7:**
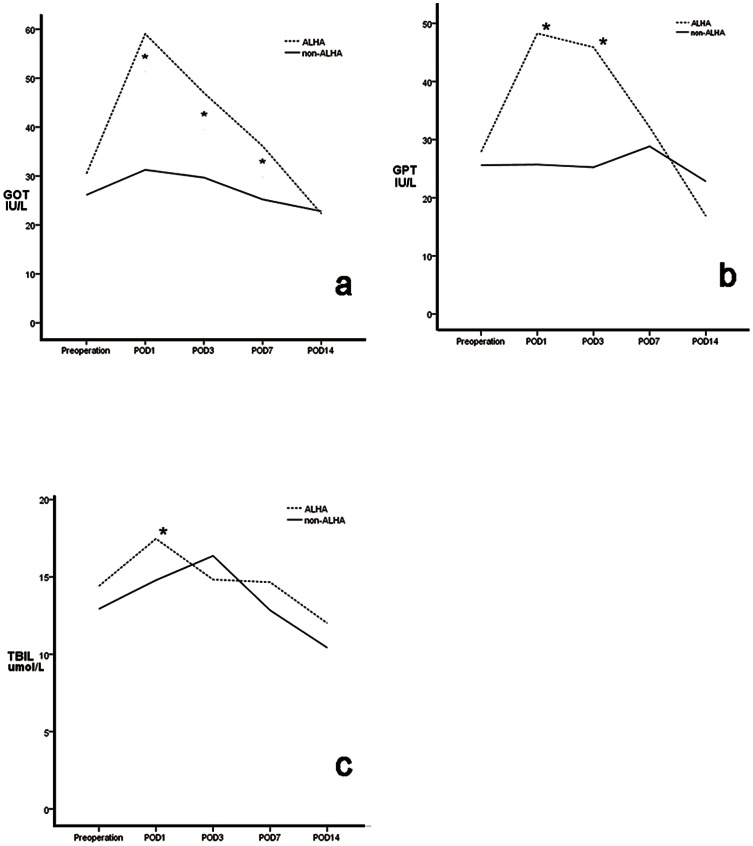
Mean GOT (a), GPT (b), and TBIL (c) concentrations in groups of patients with chronic liver diseases (CLD), with (ALHA group) and without (non-ALHA group) an accessory left hepatic artery (ALHA. Each value represents the mean. *P<0.05.

We observed postoperative liver related complication of patients within 1 month. None of the patients in either group had postoperative symptoms, such as jaundice or pruritus, related to liver dysfunction. Preoperative GOT, GPT and TBIL concentrations were similar in the two groups, as were TBIL concentrations on postoperative days 1,3, and 7 (P>0.05 each). However, GOT and GPT activities were higher in the ALHA than in the non-ALHA group on days 1 and 7 (P<0.05 each). None of these three parameters differed significantly on day 14 (Fig. 5). The proportion of patients whose liver function was still impaired at postoperative 1 week were significantly lower in the non-ALHA group (33 of the 135 (24.4%) in the ALHA group vs.174 of the 1038 (16.8%) in the non-ALHA group) (P = 0.028) (Table 4). Stratified analysis showed that preoperative GOT, GPT and TBIL concentrations in patients with and without ALHA did not differ significantly between patients with and without CLD. Furthermore, in patients without CLD, these three parameters were similar on postoperative days 1, 3, 7, and 14 in patients with and without ALHA(P>0.05 each; Fig. 6, Table 5)). Among patients with CLD, GOT and GPT concentrations on days 1 and 3 and GOT concentrations on day7 were significantly higher in patients with than without an ALHA, but returned to normal on day 14, with no significant difference between patients with and without an ALHA (Fig. 7, Table 6)). Stratified analysis also showed that the proportion of patients whose liver function was still impaired at postoperative 1 week did not differ between the ALHA group and non-ALHA group both in patients without CLD (22.4% vs. 16.4%, P = 0.118) and in patients with CLD (32.1% vs. 18.7%, P = 0.103).

**Table 4:** Mean GOT, GPT, and TBIL concentrations in groups of patients with (ALHA group) and without (non-ALHA group) an accessory left hepatic artery (ALHA).

	Group	No.	GOT	P value	GPT	P value	TBIL	P value
Preoperation	Non- ALHA	1038	22.3±11.3	0.322	20.5±16.9	0.232	12.3±7.4	0.212
	ALHA	135	23.6±14.5		23.3±26.4		11.5±6.7	
POD1	Non -ALHA	1038	27.9±21.7	0.002	23.7±19.0	0.002	14.8±5.4	0.33
	ALHA	135	38.5±39.0		33.9±36.4		15.3±5.6	
POD3	Non -ALHA	1038	25.1±24.7	0.056	23.3±19.8	0.002	16.3±7.6	0.128
	ALHA	135	29.3±17.4		28.8±15.5		15.2±5.8	
POD7	Non -ALHA	1038	24.6±22.6	0.035	27.5±29.1	0.039	13.1±10.1	0.269
	ALHA	135	28.9±22.2		34.3±36.4		14.2±8.4	
POD14	Non -ALHA	1038	22.3±15.9	0.377	23.1±19.3	0.665	10.9±8.3	0.934
	ALHA	135	23.5±13.1		23.9±19.5		10.8±4.3	

POD, postoperative day; GOT, Glutamic oxaloacetic transaminase; GPT, glutamic pyruvic transaminase; TBIL, total bilirubin; *P*-values are for comparison of the ALHA and non-AHLA groups.

**Table 5:** Mean GOT, GPT, and TBIL concentrations in groups of patients without chronic liver diseases (CLD), with (ALHA group) and without (non-ALHA group) an accessory left hepatic artery (ALHA).

	Group	No.	GOT	P value	GPT	P value	TBIL	P value
Preoperation	Non- ALHA	872	21.6±9.7	0.846	19.5±15.4	0.143	12.2±7.4	0.065
	ALHA	107	21.8±9.6		22.0±27.2		11.3±5.1	
POD1	Non -ALHA	872	27.2±13.1	0.106	23.4±17.8	0.073	14.8±5.5	0.877
	ALHA	107	33.4±39.0		30.1±37.8		14.8±5.2	
POD3	Non -ALHA	872	24.2±23.0	0.671	22.9±20.5	0.480	16.3±7.6	0.235
	ALHA	107	25.2±11.0		24.4±12.7		15.4±6.0	
POD7	Non -ALHA	872	24.5±22.8	0.236	27.2±30.1	0.061	13.2±10.6	0.443
	ALHA	107	27.4±23.7		34.8±40.3		14.0±9.3	
POD14	Non -ALHA	872	22.2. ±11.7	0.188	23.2±17.7	0.238	11.0±8.7	0.592
	ALHA	107	23.8±14.5		25.7±21.3		10.5±4.4	

POD, postoperative day; GOT, Glutamic oxaloacetic transaminase; GPT, glutamic pyruvic transaminase; TBIL, total bilirubin; *P*-values are for comparison of the ALHA and non-AHLA groups.

**Table 6:** Mean GOT, GPT, and TBIL concentrations in groups of patients with chronic liver diseases (CLD), with (ALHA group) and without (non-ALHA group) an accessory left hepatic artery (ALHA).

	Group	No.	GOT	P value	GPT	P value	TBIL	P value
Preoperation	Non- ALHA	166	26.2±16.9	0.369	25.6±22.7	0.626	12.9±7.1	0.340
	ALHA	28	30.6±24.9		27.9±22.9		14.4±9.8	
POD1	Non -ALHA	166	31.3±45.2	0.003	25.7±24.4	0.001	14.8±5.0	0.049
	ALHA	28	57.8±33.0		48.3±26.5		17.5±6.6	
POD3	Non -ALHA	166	29.7±32.1	0.017	25.3±15.5^*^	0.001	16.4±7.6	0.302
	ALHA	28	45.1±26.3		45.9±13.1		14.8±4.9	
POD7	Non -ALHA	166	25.2±21.2	0.021	28.8±23.6	0.472	12.8±7.6	0.215
	ALHA	28	34.9±14.4		32.1±13.8		14.7±3.7	
POD14	Non -ALHA	166	22.8±29.4	0.961	22.8±26.2	0.234	10.4±5.5	0.141
	ALHA	28	22.5±5.6		16.9±6.5		12.0±3.6	

POD, postoperative day; GOT, Glutamic oxaloacetic transaminase; GPT, glutamic pyruvic transaminase; TBIL, total bilirubin; *P*-values are for comparison of the ALHA and non-AHLA groups.

## Discussion

Radical gastrectomy can improve the disease-free survival rate in patients with gastric carcinoma [13]. Surgeons performing these operations should be well acquainted with the normal perigastric vascular anatomy and its variations, because failure to recognize the presence of a variant vessel can result in bleeding and other complications[14], [15]. Although an ALHA is an anomaly frequently encountered within the gastrohepatic ligament, its prevalence has been found to vary widely, with most such studies involving cadavers or angiographic data of patients undergoing hepatobiliary surgery or liver transplantation. For example, an analysis of 200 cadavers found that 16 (8%) had ALHAs arising from the left gastric artery [4]. A study of 701 patients undergoing hepatobiliary surgery or liver transplantation reported that the left hepatic artery branched from the proper hepatic artery in 89% of patients, but showed an anatomical variationin 11% [16].

Few studies, however, have assessed the prevalence of ALHA through surgical dissection. We therefore retrospectively reviewed the clinical data of 1173 patients who underwent radical gastrectomy for gastric cancer. This method, in which the hepatogastric ligament was completely separated and the vessels within it were elaborately dissected during gastric cancer surgery, accurately determined the prevalence of ALHA as well as clearly showing its anatomical features. Therefore, these results may be more accurate than those obtained at autopsy. We found that the incidence of ALHA was 11.5%, suggesting that this anomaly is quite common. Embryonic research has shown a close original relationship between the liver and stomach, which simultaneously evolve from the foregut terminal [17]. Blood is supplied to the fetal liver from the common hepatic artery, the right hepatic artery originating from the superior mesenteric artery, and the left hepatic artery originating from the left gastric artery. During embryonic development, these arteries undergo constant differentiation, growth, branching and distribution to the mature organ. The ALHA corresponds to the partial or complete persistence of the fetal pattern of the left hepatic artery, making its incidence relatively high [18]–[21]. Without knowledge of its presence, general surgeons using anultrasonic scalpel or electrotome may inadvertently sever the ALHA, increasing the risks of intraoperative and postoperative hemorrhage.

It is unclear, however, whether the ALHA should be severed during radical gastrectomy. Complications have been reported following intentional or accidental division of the ALHA, including abscess formation, cholangitis, liver failure, and even liver lobe necrosis [22]–[24]. Thus in the presence of an ALHA, some authors [24] even suggested performing a prophylactic resection of the left liver lobe when extended dissection of the lesser omentum is required in gastric or esophageal resection for malignancy.

We found, however, that our groups of patients with and without an ALHA had similar intraoperative and postoperative recovery characteristics and morbidity and mortality rates. Although our findings suggest that patients with an ALHA had poorer liver function on postoperative day 7 than those without an ALHA, stratified analysis showed no significant differences in liver function in patients without CLD between patients with a severed ALHA and those without an ALHA. In patients with CLD, however, those with a severed ALHA had significantly higher liver function indices than those without an ALHA. Angiographic examinations before and after therapeutic ligation of the hepatic artery as well as corrosion-cast studies [25]–[27] have shown collateralization between the intrahepatic and adjacent arteries. Consistent arterio-arterial anastomoses have been observed between the inferior phrenic arteries and branches of the main hepatic artery, making it possible to fill the entire arterial system of the liver by injecting into the inferior phrenicartery. Three anastomotic pathways are present from the right to the left hepatic artery, through portal(hilar) anastomoses, translobar vessels, and capsular arteries. These collaterals are observed no later than 10 h after arterial ligation [28], and neither hepatic necrosis nor death from hepatic ischemia was observed following hepatic artery ligation [26]. However, since infiltration of inflammatory cells, extensive hepatocyte necrosis and proliferation of fibrous tissue reduce liver reserve function in patients with CLD, including chronic hepatitis and cirrhosis [29]–[31], these patients are less tolerable of ischemia and hypoxia than patients without CLD. Thus, severing of the ALHA can easily induce liver dysfunction in patients with CLD. Whenever possible, therefore, the ALHA should be left intact in patients with CLD undergoing radical gastrectomy, by, for example, dividing the left gastric artery distal to the origin of the ALHA. If the ALHA is severed, however, these patients should be intensively monitored and may require liver-protecting therapy.

In conclusion, ALHA is a common anomaly that was found in 11.5% of patients. It can be safely severed during radical gastrectomy in patients without CLD, but should be left intact in patients with CLD to prevent liver dysfunction. If severed in the latter, the patient should be monitored and liver-protecting therapy may be necessary. However, because this study was nonrandomized and was based on a retrospective clinical analysis, our conclusion must be confirmed by a prospective randomized study.
